# Online interventions for problem gamblers with and without co-occurring problem drinking: study protocol of a randomized controlled trial

**DOI:** 10.1186/s13063-018-2672-x

**Published:** 2018-05-25

**Authors:** John A. Cunningham, David C. Hodgins, Matthew Keough, Christian S. Hendershot, Kylie Bennett, Anthony Bennett, Alexandra Godinho

**Affiliations:** 1 Centre for Addiction and Mental Health, 33 Russell St., Toronto, ON M5S 2S1 Canada; 2 Department of Psychiatry, University of Toronto, Toronto, Canada; 3 Austalian National University, Canberra, Australia; 4 Department of Psychology, University of Calgary, Calgary, Canada; 5 Department of Psychology, University of Manitoba, Winnipeg, Canada; 6 eHub Health Pty Ltd, Collector, Australia; 7 Dalla Lana School of Public Health, University of Toronto, Toronto, Canada

**Keywords:** Clinical trial, Randomized controlled trial, Brief interventions, Gambling disorders, Problem gambling, Comorbidity, Problem drinking, Internet intervention, Trial protocol

## Abstract

**Background:**

The current randomized controlled trial seeks to evaluate whether providing access to an Internet intervention for problem drinking in addition to an Internet intervention for problem gambling is beneficial for participants with gambling problems who do or do not have co-occurring problem drinking.

**Methods:**

Potential participants will be recruited online via a comprehensive advertisement strategy, if they meet the criteria for problem gambling. As part of the baseline measures, problem drinking will also be assessed. Eligible participants (*N* = 280) who agree to partake in the study and to be followed up for 6 months will be randomized into one of two versions of an Internet intervention for gamblers: an intervention that targets only gambling issues (G-only) and one that combines a gambling intervention with an intervention for problem drinking (G + A). For problem gamblers who exhibit co-occurring problem drinking, it is predicted that participants who are provided access to the G + A intervention will demonstrate a significantly greater level of reduction in gambling outcomes at 6 months compared to those provided access to the G-only intervention.

**Discussion:**

This trial will expand upon the current research on Internet interventions for addictions and inform the development of treatments for those with co-occurring problem drinking and gambling.

**Trial Registration:**

ClinicalTrials.gov, NCT03323606. Registered on 24 October 2017.

**Electronic supplementary material:**

The online version of this article (10.1186/s13063-018-2672-x) contains supplementary material, which is available to authorized users.

## Background

A challenge for addressing problem gambling, as with many addictions, is that the majority of people with gambling concerns never seek treatment [1–3]. To address these unmet needs, self-directed treatments have been developed, with the initial efforts focusing on bibliotherapy, and more recently, on Internet interventions. While there is a fairly strong evidence base for bibliotherapy [4–7], the evidence on Internet interventions is still emerging [8–10].

Parallel to the development of self-help interventions for problem gamblers has been the growing recognition that people experiencing gambling problems often experience other, co-occurring mental health concerns [11–16], including problem drinking [13, 14, 17–20]. An important issue to address in delivering self-help interventions (and, indeed, the delivery of face-to-face therapy) is whether there is an advantage in simultaneously providing interventions for mental health concerns alongside the provision of a gambling intervention, in particular among people who are specifically seeking help for their gambling concerns.

This protocol outlines the second of two randomized controlled trials (RCTs) addressing this question. The first trial examined the benefits of combining a gambling Internet intervention with an already validated online intervention for mood and anxiety [9]. The current trial uses a similar approach to test the benefits of combining the same online gambling intervention with a brief intervention for problem drinkers.

### Major research questions

The proposed trial will compare the efficacy of two Internet interventions: an intervention that just targets gambling issues (G-only) versus one that contains an intervention for problem drinking in addition to an intervention for gamblers (G + A). Outcome variables for gambling will measure the reduction in gambling severity and frequency of gambling. The outcome for a reduction in alcohol use will be measured by the reduction in the frequency of drinking during the past week. The primary hypotheses are:

- **Hypothesis 1:** For problem gamblers with co-occurring problem drinking, it is predicted that participants provided access to the G + A website will display significantly reduced gambling outcomes at the 3- and 6-month follow-ups compared to those provided access to the G-only website. For problem gamblers without co-occurring problem drinking, there will be no significant difference between those provided access to the G-only and G + A websites at the 3- and 6-month follow-ups.
- **Hypothesis 2:** For problem drinkers, it is predicted that participants provided access to the G + A website will display significantly reduced drinking outcomes at the 3- and 6-month follow-ups compared to those provided access to the G-only website. For participants who are not problem drinkers, there will be no significant difference in drinking between those provided access to the G-only and G + A websites at the 3- and 6-month follow-ups.
- **Hypothesis 3:** Problem gamblers with co-occurring problem drinking who receive the G + A intervention and reduce the amount they drink between the baseline and 3-month follow-ups will display significantly improved gambling outcomes at the 6-month follow-up compared to problem drinkers who receive the G + A intervention but experience no decrease in their drinking.

Follow-ups at 3 and 6 months were chosen as it is unknown whether the intervention will have the predicted impact, and we wished to optimize the possibility of finding an effect by employing a short-term follow-up.

## Methods/design

### Participants

Participants will be recruited using a comprehensive online and in-print advertisement strategy targeting Manitoba residents (e.g., local newspaper ads, social media ads on Facebook, and website ads on Yahoo and Google AdWords restricted to Manitoba residents). Advertisements will target gamblers who self-identify as being concerned about their gambling and are interested in participating in a study that aims to “find ways to help people who are worried about their gambling.” Prospective participants, 18 years or older who report gambling in the past 30 days, will be directed to complete a brief web-based screener that assesses problem gambling severity. The eligibility of individuals will be determined by their willingness to be followed up, and a current score of 3 or more on the Problem Gambling Severity Index (PGSI) [21]. Prior treatment access will be measured; however, it will not be used to determine eligibility as the intent of this trial is to evaluate the impact of the interventions within a real-world community setting. Similarly, co-occurring depression, anxiety, and illicit drug consumption will be measured but will not be used as exclusion criteria. Finally, while recruitment is not specifically targeted to attract problem drinking, it is anticipated that the trial will contain participants with and without co-occurring problem drinking. Based on the frequency of co-occurring problem drinking and gambling in the general population, it is estimated that approximately 50% of the sample will have co-occurring problem drinking [14, 22]. Participants in the first trial (examining the impact of combining an Internet intervention for gambling with one for mood and anxiety) will not be eligible to participate in the current trial.

### Study design and procedures

**Fig. 1:**
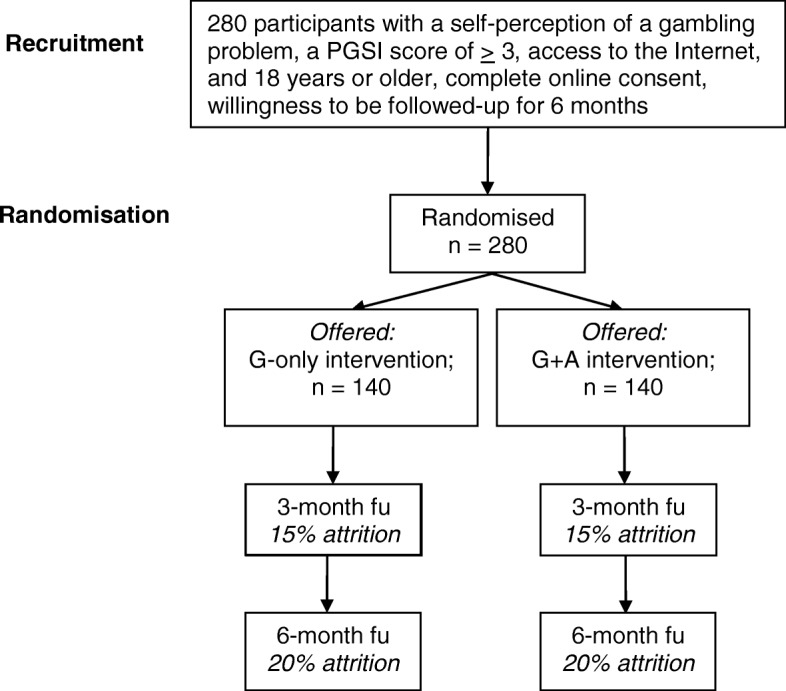
Overview of the proposed intervention trial. fu follow-up, PGSI Problem Gambling Severity Index

The proposed study is a two-arm double-blinded parallel-group RCT comparing two online interventions over a 6-month period: an intervention targeting gambling issues only versus one that contains interventions for hazardous alcohol consumption. Potential participants, who respond to the advertisement by clicking on the link or visiting the website, will be provided first with a brief description of the study prior to completing an eligibility screener. Individuals who are found to be eligible will be asked to provide contact information in the form of an email address. Subsequently, potential participants will be sent an email with a link to an online consent form. Participants will be asked to provide their telephone number and mailing address as additional contact information, as well as to provide permission for study staff to contact them via phone or mail for follow-up surveys if correspondence by email is unsuccessful. Those who complete the consent form and provide a real postal address will be sent a link to complete the baseline questionnaire and will be randomized into one of the two experimental conditions. Randomization will be stratified on age, sex, and prior use of treatment using an automatized algorithm to ensure a random distribution of prognostic indicators at baseline. To reduce attrition in the study, those who complete the baseline questionnaire and access the online intervention will receive a $10 Amazon.ca gift certificate. At 3 and 6 months post-randomization, all participants will receive an email request to complete a follow-up survey in the form of a hyper-linked web address. In addition, up to two email reminders for follow-up survey completion will be sent to participants to promote retention in the study. Furthermore, participants who complete the 3-month follow-up will be sent a $20 gift certificate and those who complete the 6-month follow-up will be sent an additional $30 gift (i.e., a total honorarium of $60 for each participant including the amount given at the baseline). Lastly, participants in both the G-only and the G + A interventions will receive an additional three emails encouraging the use of each respective intervention. See Fig. 1 for a Consort diagram summarizing the study design.

### Ethical approval

This study, including methods and design, has been approved by the standing ethics review committee of the Centre for Addiction and Mental Health (CAMH).

### Interventions

#### G-only

The gambling only Internet intervention will consist of self-change tools developed by Hodgins et al. [23] that have been adapted to an online interface. Three separate trials have demonstrated the significant impact of these tools on gambling [4, 6, 7], and their successful use within an online format has also been previously documented [8, 9]. The intent of these tools is to provide individuals with both behavioral and cognitive strategies that assist in the recovery from or a reduction in gambling.

#### G + A

The gambling plus alcohol intervention will consist of the G-only intervention as well as access to a study specific version of Check Your Drinking (CYD). The CYD contains a brief 18-item screener that has been designed to assess the quantity and frequency of drinking, and the severity of hazardous drinking [24]. Following the completion of the 18-item screener, the user is provided with a personalized report that contains normative feedback (i.e., it compares the person’s drinking with the drinking of others in the general population of the same age, sex, and country of origin, such as Canada, the U.S.A., and the U.K.). The CYD has been subjected to five randomized controlled trials from two independent research groups, all of which displayed its significant impact by reducing hazardous alcohol consumption compared to controls [24–28].

Both versions of the website contain a link to a page listing resources to contact in case of a crisis.

### Measures

**Fig. 2:**
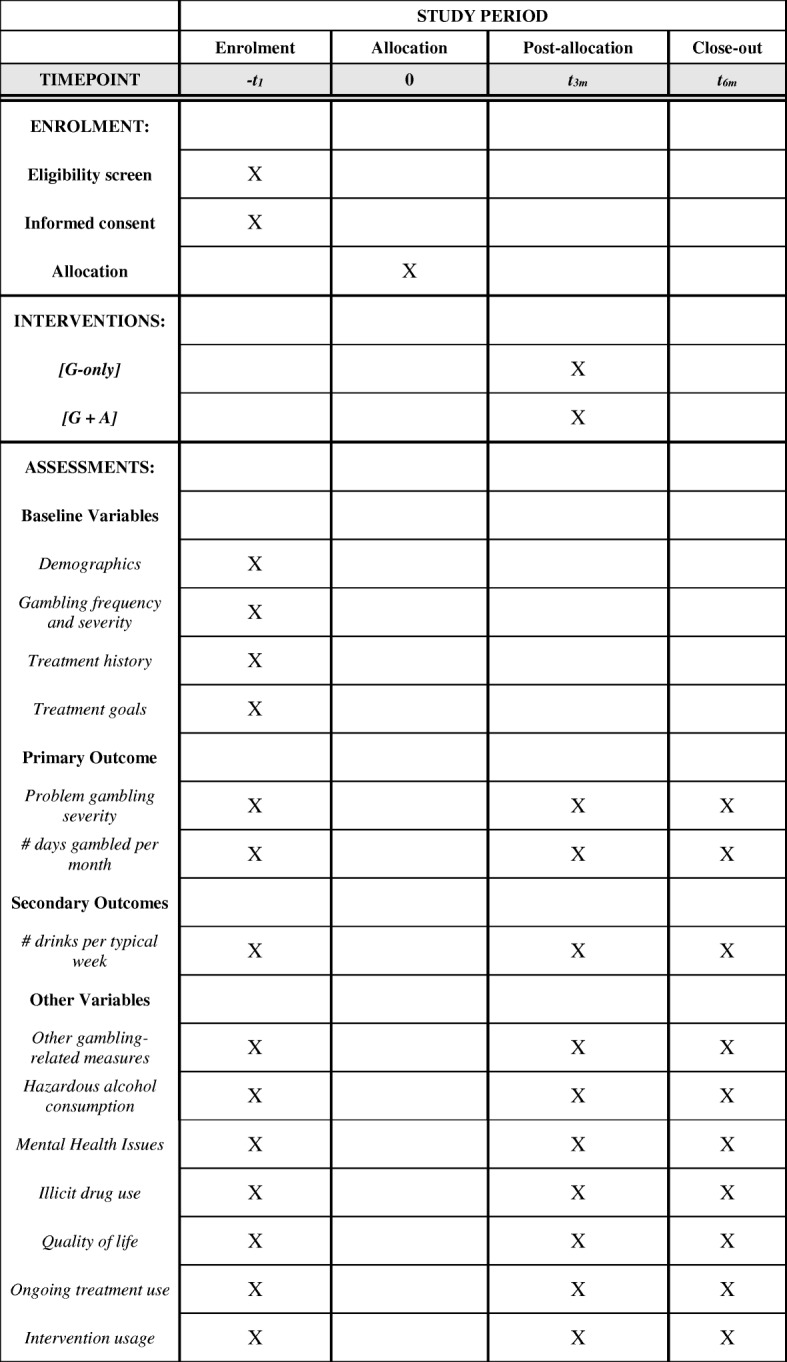
Study assessment points and instruments

Assessment points and instruments are presented in Fig. 2, and the SPIRIT checklist is available as Additional File 1.

#### Baseline survey

The baseline survey will assess demographic characteristics (e.g., age, sex, education, marital status, income, and employment status), clinical characteristics (such as gambling frequency and severity, prior treatment use, problem drinking, illicit drug use in the past year, mental health issues, i.e., depressive/anxiety symptoms and general psychological distress), and quality of life (i.e., using the World Health Organization Quality of Life 8-item survey [29]).

The severity of problem gambling will be measured using the past year PGSI and the past 3-month version of the NORC DSM-IV Screen for Gambling Problems (NODS), which indicates DSM-IV defined severity [30, 31]. The NODS has been previously employed within a 1-year follow-up study to assess brief treatment and its utility as an outcome measure [32]. Additionally, the mean number of days gambled in the past 3 months, the amount spent on gambling, and forms of gambled engaged in will be assessed. The use of treatment services for gambling concerns will be measured by asking participants to identify services accessed from a comprehensive list of treatment forms, as used in previous studies [4, 6]. Lastly, participants will also be asked to identify a treatment goal (quit or reduced gambling) and their perceived success in achieving that goal over the following 3 and 6 months on a Likert scale ranging from 0 “not at all” to 10 “extremely.” The primary outcome variables for gambling will be the NODS and number of days gambled.

Hazardous alcohol consumption will be measured using a past 3-month version of the Alcohol Use Disorders Identification Test, with the frequency of heavy drinking occasions modified for female and male participants (i.e., five or more drinks for males and four or more drinks for females) [33]. A score of 8 or more will indicate problem drinking. The primary outcome variable for alcohol consumption will be number of drinks consumed in a typical week.

The severity of depressive and anxiety symptoms will be measured using the Patient Health Questionnaire 9-item scale (PHQ-9) [34] and the Generalized Anxiety Disorder 7-item scale (GAD-7) [35], respectively. In addition, general psychological distress will be measured using the Kessler 10 questionnaire (K10), as this measure has been found to be responsive to change over time. The K10 has been well validated and is appropriate for self-administration, as it is brief, simple, and easy to comprehend [36, 37].

#### Follow-up surveys

The 3- and 6-month surveys will employ the same measures as those assessed at baseline using a past 3-month timeframe.

### Use of interventions

A count of participant logins into the intervention, as well as the various web pages accessed within the G-only intervention (as well as use of the CYD), will be available. These data will be used to investigate whether participants’ degree of involvement with the intervention is associated with their success at recovering from gambling or reducing gambling severity. The degree of G-only intervention use by participants will be operationalized according to the previously recommended methods [38, 39]. The number of times a participant accesses the site, the number of tools the participant uses (as assessed by page views, form completions, etc.), and the length of involvement with the site (e.g., use of the site over time) will be recorded.

### Power analysis

Efforts will be made to recruit a total sample of 280 participants, and we anticipate that 224 participants will be successfully followed up at 6 months (i.e., estimated 20% attrition based on previous research of online interventions, albeit targeting hazardous alcohol use) [40]. This sample size will provide sufficient power to conduct the proposed statistical analyses necessary to test the aforementioned hypotheses. Based upon previous data collected by Hodgins et al. [4, 6] on gambling frequency and NODS scores, and assuming that a correlation of 0.5 exists between baseline and follow-up measures, then power = .0.80 and α = .05. This sample size is sufficient to detect a difference of at least two gambling days per month between both experimental conditions in both the 3- and 6-month follow-up periods. Smaller differences in gambling days per month may not be clinically meaningful. Similarly, the proposed sample size will also be sufficiently powered to detect a 1-point difference of NODS scores at the 6-month follow-up. Calculations are based upon a repeated measures ANOVA model (G.Power 3.1.9).

### Data analysis

The two primary hypotheses of this study, which focus on comparing outcomes between groups for gambling and drinking respectively, will be analyzed using linear mixed-effect models with random intercepts. This will allow us to use all available participant data within the models, employing a restricted maximum likelihood to handle missing data. Separate analyses will be conducted for each primary outcome variable (i.e., NODS score, mean number of day gambled in the past month, and, for drinking, the number of drinks consumed in a typical week). For secondary analyses, mixed-effects models will be conducted to examine the effect of moderators on primary variables, that is, additional fixed-effect interactions will be included in exploratory models (e.g., extent of intervention use, participants’ sex, severity of gambling and drinking at baseline, and co-occurrence of mental health concerns). In addition, secondary analyses will also examine predictors of treatment use, as well as the effect of accessing treatment on primary outcomes. The third hypothesis, comparing the potential moderating effect of reductions in drinking on gambling severity, will be examined using Hayes’s Process macro. This method uses a state-of-the-art regression-based conditional process approach [41] to estimate covariances, variances, and means. Missing data will be handled using a maximum likelihood approach. Finally, we will examine whether changes in quality of life are related to improvements in gambling outcomes.

## Discussion

Many problem gamblers are also problem drinkers [13, 14, 17–20], with a lifetime prevalence as measured by nationally representative samples ranging from 45% to 73% [14, 22]. Heavy drinking often occurs while problem gamblers are engaging in gambling activities [42], resulting in increased risky gambling behavior [43–45]. Further, co-occurring problem drinking negatively impacts the treatment outcomes of problem gamblers (see the systematic review by Merkouris et al. [46]). Targeting problem drinking by problem gamblers may have the dual benefits of reducing both the problem drinking itself and of acting as a mediator for reductions in problem gambling behavior.

The proposed study seeks to determine whether providing simultaneous Internet interventions for gambling and drinking is of benefit for those with these co-occurring problems. More specifically, it will evaluate whether there is an incremental benefit to providing access to a problem drinking Internet intervention (G + A intervention) in addition to an Internet intervention for problem gambling (G-only intervention) for individuals with gambling problems who do or do not have co-occurring problem drinking. A limitation of the current trial is its short follow-up period (6-month follow-up). If the trial finds that the addition of a brief alcohol intervention to an online gambling intervention is beneficial at this time point, then future research examining any sustained impact of the intervention would be merited. The results of this study will enhance our understanding of Internet interventions for addictions in general, as well as inform how treatments can be developed and matched to the care of and needs for those with co-occurring problem gambling and drinking.

### Trial Status

Protocol version: 1.

Date recruitment began: 20 November 2017.

Approximate date recruitment will be completed: December 2018.

## Additional file

Additional file 1: SPIRIT 2013 Checklist: Recommended items to address in a clinical trial protocol and related documents. (DOC 121 kb)

## Abbreviations

CAMH: Centre for Addiction and Mental Health
CYD: Check Your Drinking
DSM-4: Diagnostic Statistical Manual Version 5
G + A: G-only intervention plus access to Check Your Drinking, an Internet intervention for hazardous drinking
GAD-7: Generalized Anxiety Disorder 7-item scale
K10: Kessler Psychological Distress Scale
NODS: NORC Diagnostic Screen for Gambling Problems
PGSI: Problem Gambling Severity Index
PHQ-9: Patient Health Questionnaire 9-item scale
RCT: Randomized controlled trial
